# Knockdown of CENPK inhibits cell growth and facilitates apoptosis via PTEN‐PI3K‐AKT signalling pathway in gastric cancer

**DOI:** 10.1111/jcmm.16850

**Published:** 2021-08-12

**Authors:** Shusheng Wu, Lulu Cao, Lihong Ke, Ying Yan, Huiqin Luo, Xiaoxiu Hu, Jiayu Niu, Huimin Li, Huijun Xu, Wenju Chen, Yueyin Pan, Yifu He

**Affiliations:** ^1^ Anhui Provincial Hospital Cheeloo College of Medicine Shandong University Jinan Shandong China; ^2^ Department of Medical Oncology The First Affiliated Hospital of USTC Division of Life Sciences and Medicine University of Science and Technology of China Hefei Anhui China; ^3^ Department of Medical Oncology Anhui Provincial Hospital Hefei Anhui China

**Keywords:** CENPK, gastric cancer, PI3K‐AKT signalling pathway, proliferation, PTEN

## Abstract

Previous studies have indicated that centromere protein K (CENPK) is upregulated in several cancers and related to tumorigenesis. Nevertheless, the potential function of CENPK in gastric cancer (GC) remains unknown. Here, we investigated the function of CENPK on oncogenicity and explored its underlying mechanisms in GC. Our results showed that CENPK was dramatically overexpressed in GC and was associated with poor prognosis through bioinformatics analysis. We demonstrated that CENPK is upregulated in GC tissues and cell lines. Moreover, knockdown of CENPK significantly inhibited proliferation in vitro and attenuated the growth of implanted GCs in vivo. In addition, CENPK silencing induced G1 phase cell cycle arrest and facilitated apoptosis of GC cells. KEGG pathway analysis indicated that the PI3K‐AKT signalling pathway was considerably enriched. Knockdown of CENPK decreased the expression of PI3K, p‐Akt (Ser437) and p‐GSK3β (Ser9) in GC cells, and increased the expression of PTEN. In conclusion, this study indicated that CENPK was overexpressed in GC and may promote gastric carcinogenesis through the PTEN‐PI3K‐AKT signalling pathway. Thus, CENPK may be a potential target for cancer therapeutics in GC.

## 1 INTRODUCTION

Globally, the incidence of gastric cancer (GC) ranks fifth and the mortality rate ranks fourth.^1^ One million incident cases are estimated annually, and Eastern Asia has the highest incidence.^2^ China has 478,508 newly diagnosed cases of GC and 373,789 cancer‐related deaths in 2020.^1^ Although the proportion of early GC in northern China increased from 10.0% to 15.5%, there were still 28.1% initial diagnoses of TNM stage IV GC.^3^ The most common therapy currently for GC is surgical resection; however, distant metastasis requires chemotherapy. Despite the development of combined treatment strategies and a deep understanding of the pathogenesis of GC, the mortality rate of patients with GC is relatively high.^4^ Therefore, there is an urgent need to explore new molecular markers and discover innovative methods for treating GC.

During mitosis, accurate chromosome separation is fundamental for maintaining the number of diploid chromosomes.^5^ Most cancer cells are aneuploid and have unstable chromosomes, which means that chromosomes increase or decrease with each mitosis.^6^ Increasing evidence suggests that dysregulation or dysfunction of centromeres represents a possible source of chromosomal instability and aneuploidy production and promotes the development of cancer.^7^, ^8^ Centromere protein K (CENPK), also known as AF‐5alpha, Solt, ICEN37, FKSGl4 and P33, is one of the centromere proteins (CENPs), which is located on chromosome 5q12.3.^9^ CENPK is a component of the CENPA‐CAD (distal nucleosome) complex, involved in the assembly of centromere proteins, mitotic processes and chromosome separation. It may be related to the newly synthesized centromere protein A (CENPA) binding to the centromere through the interaction with the CENPA‐nac complex and cooperates with KNL1 (Kinetochore Scaffold 1) to recruit the NDC80 (Kinetochore Complex Component) complex to the external centromere.^10^ CENPA is one of the earliest centromere components discovered in humans and is involved in the development of several human malignancies.^11^ Centromere proteins related to the development of malignant tumours include CENPA,^11^, ^12^ CENPE,^13^ CENPH^14^ and CENPK.^15^ These proteins play a role in many important cellular activities, such as gene transcription^12^ and cell cycle process regulation,^13^ and are closely linked to apoptosis and tumour invasion.^14^ Nevertheless, the relevant regulatory mechanisms remain unknown.

CENPK is associated with malignant progression and is markedly upregulated in ovarian cancer,^15^ triple‐negative breast cancer^16^ and liver cancer.^17^ As a less studied member of the centromere protein family, CENPK was associated with tumorigenesis, implying that CENPK might serve as a prognostic target for cancers. To the best of our knowledge, there are no reports of CENPK contributing to the occurrence or progression of GC or the potential clinical significance of CENPK expression in GC patients. With this in mind, we were interested in studying the regulatory role of CENPK in GC. In this study, we aimed to detect the expression pattern and function of CENPK and explore the possible mechanism of CENPK in GC. In the present study, we examined the expression of CENPK in GC tissues and found that CENPK expression was significantly upregulated in GC tissues compared with adjacent normal tissues. Furthermore, we demonstrated that CENPK silencing inhibited tumorigenesis in vivo and in vitro, induced cell cycle arrest and triggered apoptosis. Meanwhile, we found that CENPK silencing resulted in the decreased expression of PI3K, p‐Akt (Ser437) and p‐GSK3β (Ser9) and the increased expression of PTEN. Based on these results, we hypothesized that CENPK is a potential oncogene in GC.

## 2 MATERIALS AND METHODS

### 2.1 Bioinformatics database prediction

We initially used the Oncomine database to predict the level of CENPK mRNA expression in GC and normal gastric tissues. The online database Gene Expression Profiling Interactive Analysis (GEPIA)^18^ and UALCAN cancer database^19^ were used to further forecast the expression level of CENPK mRNA in GC and normal gastric tissues. Subsequently, we logged in to the official portal GDC ( https://portal.gdc.cancer.gov/) of The Cancer Genome Atlas (TCGA) to download relevant data for GC. A total of 404 cases containing CENPK gene expression information were downloaded. The R software (3.5.3) was used with the "edgeR" and "DSEeq" packages to normalize the data. At the same time, the clinicopathological information, including survival time, was screened from the downloaded data. The data containing the CENPK gene expression information and clinicopathological characteristics were combined to obtain a total of 263 cases for further analysis. Finally, all patients were divided into the CENPK high‐expression group or the CENPK low‐expression group by the "surv_cutpoint" function. The patient survival curve was drawn using the Kaplan‐Meier method, and Cox multivariate survival analysis was performed. The detailed clinicopathological characteristics of the 263 patients are shown in Table S1.

The function of CENPK and the genes significantly related to CENPK alterations were predicted through the Gene Ontology (GO) and Kyoto Encyclopedia of Genes and Genomes (KEGG) analysis. The potential molecular function of CENPK can be reversed through the co‐expression genes most relevant to CENPK expression, a method known as the guilt of association.^20^ The CENPK gene was analysed in batches with other encoded genes to obtain the correlation coefficient and *p*‐value. Genes with a *p*‐value < 0.05 and the top 500 in the absolute value of the correlation coefficient were screened for enrichment analysis (Table S2). GO enrichment analysis predicted the functional roles of target host genes from three fields: biological processes, cell composition and molecular function. Subsequently, the “cluster profiler”^21^ package was used to analyse and visualize gene ontology in the CC, MF and BP categories using R software. Furthermore, the KEGG pathway enrichment analysis was conducted, where adjusted *p* < 0.05 was considered statistically significant.^22^

### 2.2 Clinical specimens

Twenty pairs of paraffin tissues, including GC tissues and adjacent normal tissues, from patients undergoing gastrectomy in 2019 were collected from the First Affiliated Hospital of the University of Science and Technology of China. The inclusion criteria were postoperative pathological diagnosis of gastric adenocarcinoma and not receiving any anticancer treatment before surgery. The exclusion criterion was that only one postoperative paraffin specimen was available. This study was approved by the Ethics Committee of The First Affiliated Hospital of University of Science and Technology of China (West District, Hefei, China). The ethics committee waived the need for written informed consent because the study was conducted using residual samples from previous clinical diagnoses, the risks of the study did not exceed minimal risks, and the waiver of informed consent would not adversely affect the subjects.

### 2.3 Immunohistochemistry

Tissues were altered with 4% paraformaldehyde at 4°C for 48 h, and paraffin was cut into 5 μm thick segments. After sectioning, the slides were deparaffinized and rehydrated. For antigen retrieval, slides were warmed at the sub‐boiling point temperature using a citrate cushion (ph6. 0) for 10 min, cooled to room temperature and washed three times with PBS for 5 min each time. Endogenous peroxidase activity was blocked with immunostaining blocking solution at room temperature for 15 min, and the slides were incubated with anti‐CENPK (1:200; MyBioSource, San Diego, CA, USA) at 4°C overnight. The following day, slides were incubated with secondary antibodies (Abbkine Scientific, Wuhan, China) at 37°C for 30 min. Following diaminobenzidine (DAB) staining, haematoxylin staining was performed. A staining score lower than 4 indicated negative CENPK expression, and a score higher than 4 indicated positive CENPK expression.^23^

### 2.4 Cell lines

The human normal gastric epithelial cell line (GES‐1) and GC cell lines SGC‐7901, MGC‐803, HGC‐27 and AGS were purchased from the cell bank of the Chinese Academy of Sciences (Shanghai, China). Cells were cultured in RPMI1640 (Gibco, Carlsbad, CA, USA) supplemented with 10% foetal bovine serum (FBS, 10099141; Gibco BRL/Invitrogen, CA, USA) and incubated at 37°C with 5% CO2.

### 2.5 Quantitative real‐time polymerase chain reaction (qRT‐PCR)

Total RNA was extracted from cells using TRIzol (Life Technologies, CA, USA). Two micrograms of total RNA from each test was reverse‐transcribed using M‐MLV‐RTase (Promega, Madison, WI, USA), according to the manufacturer's guidelines. *q*reverse transcriptase‐polymerase chain reaction (qRT‐PCR) was performed in a Bio‐Rad Real‐Time PCR detection system utilizing the SYBR premix ex taq (Takara Bio, Dalian, China). The primers used were as follows: CENPK forward, 5'‐AGTACCTTGGGCGAGTTTCTA‐3’ and reverse, 5'‐AGGCAATTCCATTACGCAGCA‐3’; PTEN forward, 5'‐TTTGAAGACCATAACCCACCAC‐3’ and reverse, 5'‐ATTACACCAGTTCGTCCCTTTC‐3’; and GAPDH forward, 5'‐GGAAGCTTGTCATCAATGGAAATC‐3’ and reverse, 5'‐TGATGACCCTTTTGGCTCCC‐3'. Relative expression levels were calculated using the 2^−ΔΔCt^ method.

### 2.6 Lentiviral plasmids and cell transfection

Biogenetech (Shanghai, China) designed and synthesized lentiviral particles containing shRNA sequences targeting CENPK (AGTACCTTGGGCGAGTTTCTG). AGS cell was infected with CENPK‐shRNA lentiviral particles. AGS cell suspension was seeded on a six‐well plate with 5 × 10^4^ cells per well and incubated at 37°C until 30% confluence was reached. Subsequently, CENPK shRNA lentivirus (shCENPK) and negative control (shNC) were affiliated based on the multiplicity of infection. The infected cells were cultured in a CO2 incubator for 12h. After 72 h of infection, the expression of the GFP‐labelled gene was observed under a fluorescence microscope. Cells with a transfection efficiency >70% were selected for subsequent analyses. The knockdown effectiveness of CENPK in the AGS cells was verified using qRT‐PCR.

### 2.7 Proliferation assay

CENPK‐knockdown cells and NC cells were used for proliferation detection. Cell Counting Kit 8 (CCK‐8) was used to detect cell proliferation. After transfection, 1000 cells per well were placed on a 96‐well plate and preincubated overnight. Next, 10 μL of CCK‐8 reagent (BioLite Biotech, Tianjin, China) reagent was infused into each well once a day for four days. After 2 h of incubation, the optical density of each well was determined at 450 nm using a microplate reader.

### 2.8 Apoptosis and cell cycle distribution

Apoptotic cells were analysed using flow cytometry. After 72 h of transfection, AGS cells were collected and washed twice with ice‐cold PBS. Apoptotic cells were assayed by staining with annexin V‐APC (AAT Bioquest, Sunnyvale, CA, USA). Apoptosis was simultaneously assayed by caspase 3/7 (AAT Bioquest), which was designed to assay cell apoptosis by caspase 3 activation. Briefly, cells were prepared by adding 10 µL/well of 10X test compounds, and then an equal volume of caspase 3/7 working solution was added and incubated at room temperature for 1 h following the manufacturer's protocol. A fluorescence microplate reader was used to measure the fluorescence intensity at Ex/Em = 350/450 nm (cut‐off = 420 nm).

Adherent cells were collected and washed with pre‐cooled D‐Hanks. Cells for cycle distribution were settled with pre‐cooled 75% ethanol at 4°C for at least 1 h, then incubated with 100 μL RNase A (Fermentas, Shanghai, China) at 37°C for 30 min and stained with propidium iodide (Sigma, St. Louis, MO, USA). The suspension was then subjected to flow cytometry analysis.

### 2.9 Animal experiments

Twenty female BALB/c nude mice (4 weeks old) were purchased from Shanghai Lingchang BioTech Co., Ltd. (Shanghai, China). The concentration of luciferase‐labelled AGS cells that were transfected with shCENPK or shNC in the logarithmic phase was adjusted to 2 × 10^7^ cells/ml in PBS solution. Two hundred microlitres of the cell suspension were injected subcutaneously into female BALB/c nude mice. The formula: volume = π/6 × L × W × W was used to calculate tumour volumes, where W and L represent the short diameter and the long diameter of the tumours, respectively. Subsequently, the measurements were performed three times a week. After 28 days of subcutaneous injection, the experimental animals were euthanized with an overdose of 2% pentobarbital sodium, and tumour tissues from the sacrificed mice were removed.

Mice were intraperitoneally injected with D‐luciferin (15 mg/ml, Qianchen, Shanghai, China) at a dose of 10 µl/g before anaesthetization with 0.7% pentobarbital sodium the day before euthanasia. Images were acquired and analysed using a Caliper IVIS Lumina II (PerkinElmer, Waltham, MA, USA). The total flux of the region of interest (ROI) was recorded in photons/s after 5 s of exposure for each animal. All animal studies were performed in accordance with the guidelines issued in the Guide for the Care and Use of Laboratory Animals of the National Institutes of Health.

### 2.10 Western blot assay

Western blot analysis was performed as previously described.^24^ The primary antibodies included p‐Akt (Ser473) (CST; 4060), GAPDH (Abcam; ab181602), cyclin D1 (Abcam; ab16663), p‐GSK3β (Ser9) (CST, 5558), CDK4 (Abcam; ab199728), P21 (Abcam; ab109199), GSK3β (CST, 12456), P27 (Abcam; ab193379), PI3K (Abcam; ab86714), Akt (CST, 4691) and PTEN (ProteinTech; 10047‐1‐AP). GAPDH was used to homogenize the samples.

### 2.11 Statistics

The correlation between CENPK expression levels and clinicopathological parameters was analysed using the Wilcoxon rank‐sum test. Kaplan‐Meier survival analysis was used to compare the overall survival (OS) between the two groups. Each experiment was independently conducted in triplicates. Continuous data were expressed as mean ± standard deviation (*SD*) using two‐way analysis of variance or *t* test. Statistical analyses were performed using GraphPad Prism 8 and SPSS 24.0. The significance level was characterized by the probability *p < 0.05, **p < 0.01, ***p < 0.001 and ****p < 0.0001.

## 3 RESULTS

### 3.1 Overexpression of CENPK mRNA levels in GC predicted by bioinformatics

**FIGURE 1:**
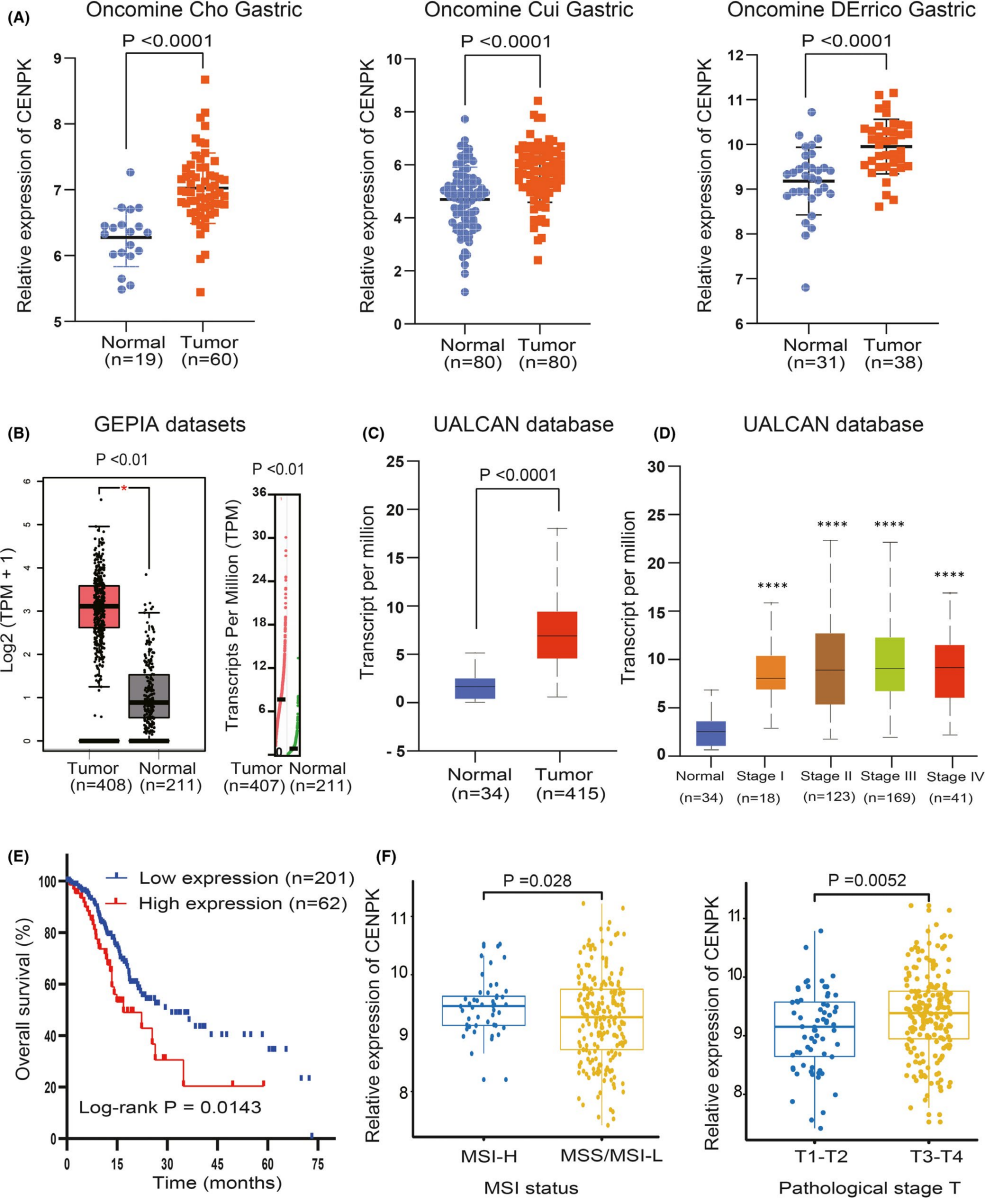
CENPK is upregulated in GC and correlates with overall survival. A, All three gastric data sets (Cho gastric, Cui gastric and DErrico gastric) in the Oncomine database showed significantly higher expression levels of CENPK mRNA in GC tissues than in normal gastric tissues (all *p* < 0.0001). B, According to the GEPIA database, the transcripts per million (TMP) and log2 (TMP + 1) levels of CENPK in GC were significantly higher than those in normal gastric tissues (all *p* < 0.01). C and D, In the Ualcan database, CENPK was significantly overexpressed in GC compared with normal gastric tissues (*p* < 0.0001). Significantly higher expression of CENPK was seen in different stages of GC when compared with normal gastric tissue (all *p* < 0.0001). However, CENPK expression was not statistically different between the different stages of GC. E, CENPK high expression was correlated with a poor survival rate of GC patients (*n* = 263, *p *< 0.05). F, CENPK expression was significantly increased in patients with microsatellite instability‐high (MSI‐H) (*p* = 0.028) and pathological stage tumour depth T3‐T4 (*p* = 0.0052). ****p < 0.0001

First, we used three gastric datasets (Cho gastric,^25^ Cui gastric,^26^ DErrico gastric^27^) in the Oncomine database to detect the differences in the expression of CENPK between GC and normal gastric tissues. Significantly increased expression of CENPK was observed in GC vs. normal gastric tissues in all three datasets (*p* < 0.0001, Figure 1A). The GEPIA also indicated that the transcripts per million (TMP) and log2 (TMP +1) levels of the CENPK gene in GC were significantly higher than those in normal gastric tissues (all *p* < 0.01, Figure 1B). This result is consistent with that of the Ualcan database. In the Ualcan database, which is based on TCGA samples, CENPK was overexpressed in GC compared with normal gastric tissues (*p* < 0.0001, Figure 1C). According to TNM staging, GC is classified into stages I‐IV. Significantly higher expression of CENPK was seen in different stages of GC when compared with normal gastric tissue (all *p* < 0.0001). However, CENPK expression was not statistically different between the different stages of GC compared with each other (Figure 1D).

### 3.2 Overexpression of CENPK associated with tumour progression

Two hundred and sixty‐three patients with GC in TCGA were divided into the low‐expression group (*n* = 201) and the high‐expression group (*n* = 62). We found that the OS of patients in the high‐expression group was worse than that of the low‐expression group (*p* = 0.034, Figure 1E). GC patients were classified into microsatellite stable (MSS), microsatellite highly unstable (MSI‐H) and microsatellite low unstable (MSI‐L) according to their microsatellite status. The expression of CENPK in MSI‐H was significantly higher than that in MSS/MSI‐L (*p* = 0.028, Figure 1F), and the expression of CENPK in the pathological stage tumour depth T3‐T4 was observably higher than that in T1‐T2 (*p* = 0.0052, Figure 1F). In the multivariate analysis, CENPK was independently associated with OS (*p* = 0.023, HR: 1.681; 95% CI: 1.075–2.630, Table 1).

**TABLE 1:** Multivariate analysis

Characteristics		HR (95% CI)	*p* Value
Age (years)	<65 vs. ≥65	2.088 (1.366–3.193)	0.001
Gender	Female vs. male	1.270 (0.816–1.975)	0.289
Histologic grade	Grade 1–2 vs. grade 3	1.644 (1.072–2.522)	0.023
CENPK expression	Low vs. high	1.681 (1.075–2.630)	0.023
MSI status	MSS/MSI‐L vs. MSI‐H	0.822 (0.472–1.431)	0.488
Tumour stage	Stage I–II vs. stage III–IV	1.657 (1.064–2.579)	0.025
Pathological stage T	T1–T2 vs. T3‐T4	1.218 (0.701–2.116)	0.485
Lymph node metastasis	No vs. yes	1.006 (0.481–2.106)	0.987
Distant metastasis	No vs. yes	2.688 (1.303–5.546)	0.007

Abbreviations: MSI, microsatellite instability; MSI‐H, MSI‐high; MSS, microsatellite stable; MSI‐L, MSI‐low; pathological T, pathological tumour depth.

### 3.3 Overexpression of CENPK in GC cell lines and tissues

**FIGURE 2:**
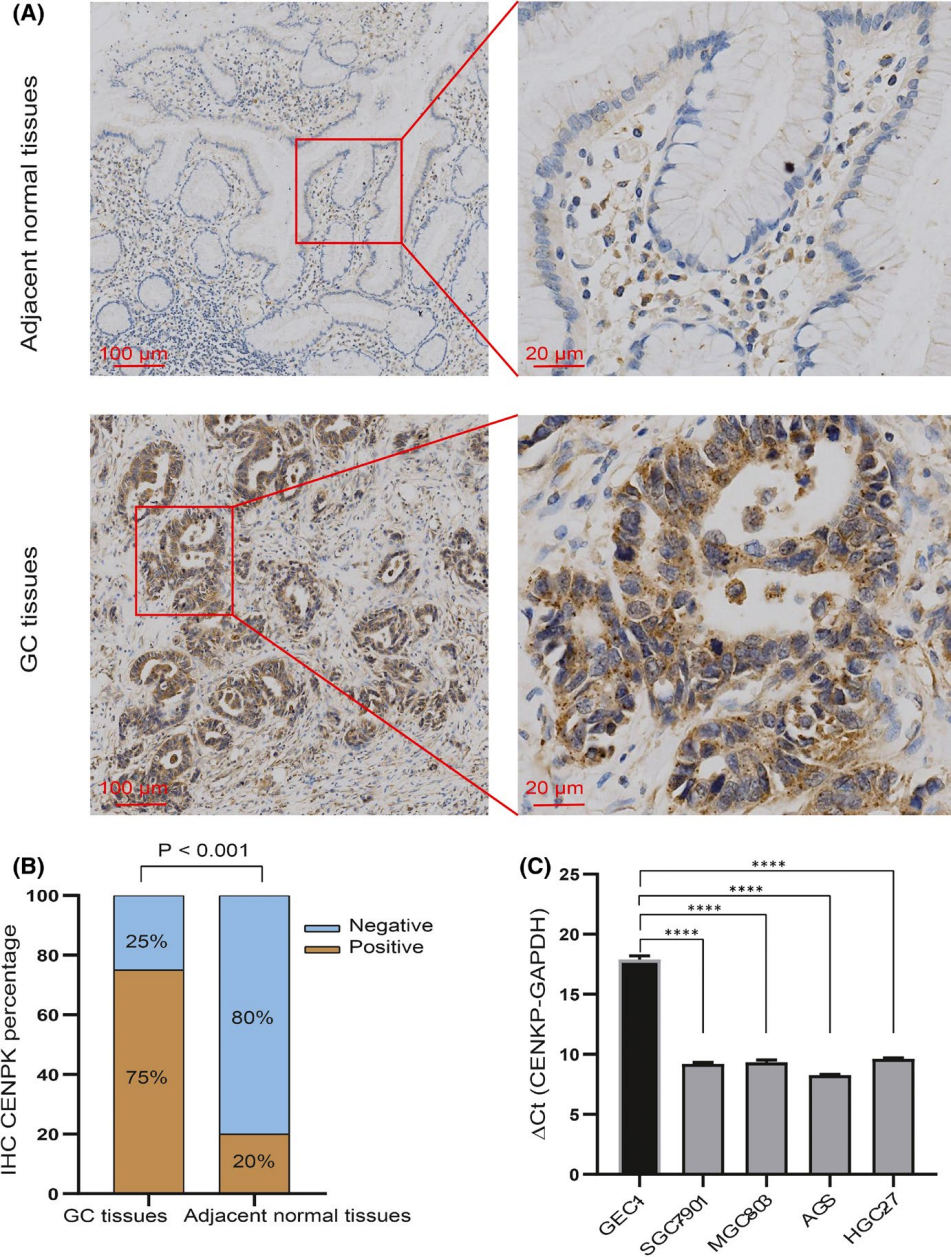
Overexpression of CENPK in GC tissues and cell lines. (A) Representative images of IHC for CENPK expression in GC tissues and adjacent normal tissues. Scale bar, 100 μm (left), 20 μm (right). B, Percentage of CENPK IHC in GC and adjacent normal tissues. C, The relative mRNA expressions of CENPK levels were detected in four kinds of GC cell lines (SGC‐7901, HGC‐27, MGC‐803 and AGS) and human normal gastric epithelial cell line (GES‐1) by qRT‐PCR. Quantitative data were shown as mean ± *SD*. ΔCt value ≤ 12, CENPK expression abundance was high; 12 < ΔCt value < 16, CENPK expression abundance was medium; ΔCt value ≥ 16, CENPK expression abundance was low. ****p < 0.0001

To verify the aforementioned predictive findings, we tested 20 pairs of paraffin samples from patients with GC by immunohistochemistry. As shown in Figure 2A, the expression of CENPK in GC tissues was significantly increased. Among 20 pairs of postoperative GC tissues, 15 cases of GC tissues were positive for CENPK staining, and five cases were negative. However, 16 cases of adjacent normal tissues were negative for CENPK, and four cases were positive (Figure 2B, *p* < 0.001). We also explored the expression of CENPK mRNA in GC cell lines, the human normal gastric epithelial cell line (GES‐1) and four types of GC cell lines (AGS, MGC‐803, HGC‐27 and SGC‐7901) were selected for qRT‐PCR analysis. Significantly increased CENPK mRNA levels were found in the four GC cell lines compared with GES1 (all *p* values < 0.0001, Figure 2C).

### 3.4 CENPK silencing inhibits cell proliferation in vitro

**FIGURE 3:**
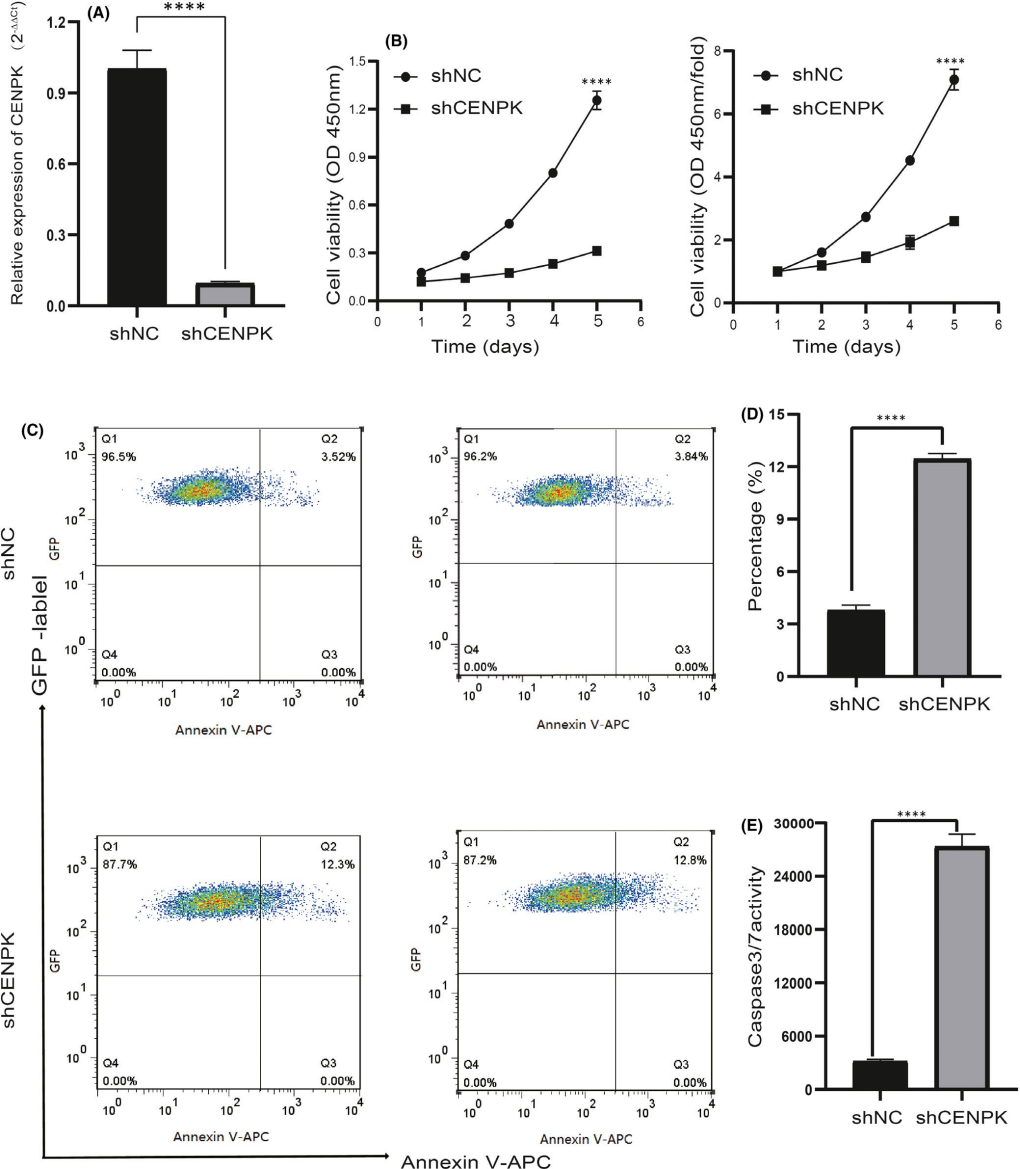
Knockdown of CENPK inhibited cell growth and promoted apoptosis in AGS cells. A, The relative mRNA expressions of CENPK after CENPK knockdown in AGS cells were identified by qRT‐PCR. B, Knockdown of CENPK inhibited cell proliferation. The OD 450nm and OD 450nm/fold values of AGS cells were detected by CCK‐8 assay. C and D, Representative images and bar plots of apoptosis rate in the AGS cells. E, Knockdown of CENPK increased the activity of caspase 3/7. ****p < 0.0001

As substantial overexpression of CENPK was observed in several GC cell lines, AGS cells were transfected with shCENPK or shNC. Figure 3A demonstrates that the introduction of a lentivirus carrying the CENPK gene resulted in a significant decrease in CENPK expression, indicating successful transfection. The CCK‐8 assay revealed that silencing CENPK significantly inhibited the proliferation of GC cells (Figure 3B).

### 3.5 CENPK silencing promotes apoptosis

To determine the effect of CENPK expression on cell survival, cell apoptosis was analysed using flow cytometry. There was a significant increase in the proportion of apoptotic cells in CENPK‐silenced cells compared with that in controls (Figure 3C‐D, *p* < 0.0001). Caspase 3/7 activity apoptosis assay was performed to monitor cell viability. Figure 3E shows that the activity of caspase 3/7 and the number of apoptotic cells distinctly increased in the shCENPK group compared with the control group (*p* < 0.0001). These data indicate that knockdown of CENPK‐affected cell survival.

### 3.6 CENPK silencing induces G1 phase arrest in GC cells

**FIGURE 4:**
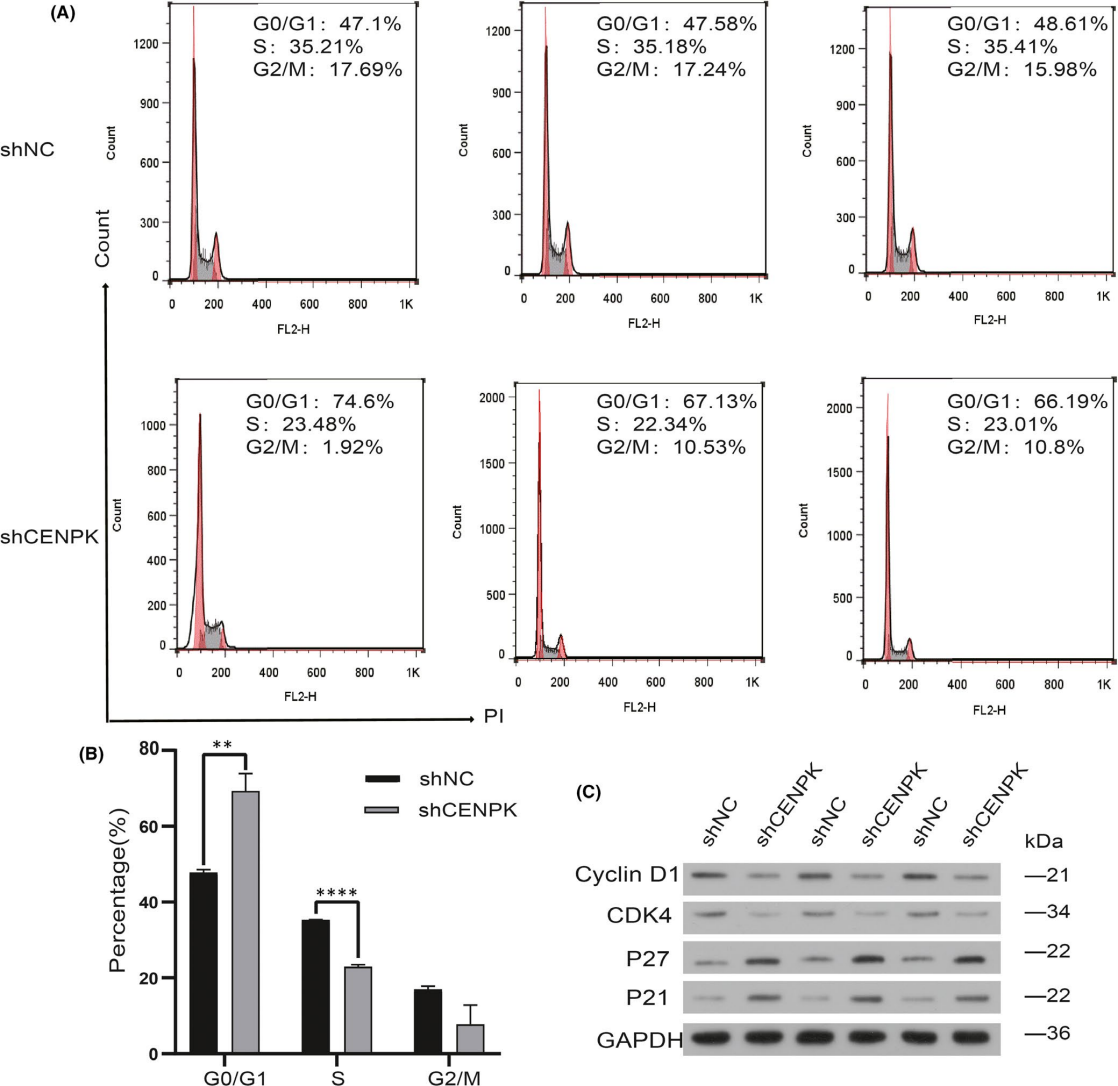
Knockdown of CENPK blocked cell cycle at the G1 phase in AGS cells. A, After transfection, flow cytometry showed that shCENPK induced accumulation in the G0/G1 phase. Each group is displayed in triplicates. B, Bar plots of flow cytometry analysis in the AGS cells. The percentages of cells at different phases are expressed as the mean ± SD of three independent experiments. C, G1 phase‐associated molecules were determined by Western blotting assay in AGS cells. **p < 0.01, ****p < 0.0001

To investigate the mechanism by which CENPK promotes tumour cell proliferation, we examined the effect of CENPK on the cell cycle using flow cytometry. Compared with the control group, the shCENPK group showed a significant increase in the proportion of G0/G1 phase cells (*p* < 0.01), a significant decrease in S phase cells (*p* < 0.0001) and a decrease in G2/M phase cells, but the difference was not statistically significant (*p* > 0.05; Figure 4A‐B). Cell cycle regulators, including cyclins, cyclin‐dependent kinases (CDKs) and kinase inhibition protein (KIP), play a vital in cell cycle progression. Thus, G1 phase‐related molecules, including cyclin D1, CDK4, p21 and p27 in AGS cells after CENPK knockdown were identified by Western blotting. Figure 4C illustrates that the expression of CDK4 and cyclin D1 protein in the shCENPK group decreased, whereas p21 and p27 were increased in AGS cells following shCENPK transfection. These results implied that knockdown of CENPK may result in cell cycle arrest in the G1 phase.

### 3.7 CENPK silencing inhibits cell growth in vivo

**FIGURE 5:**
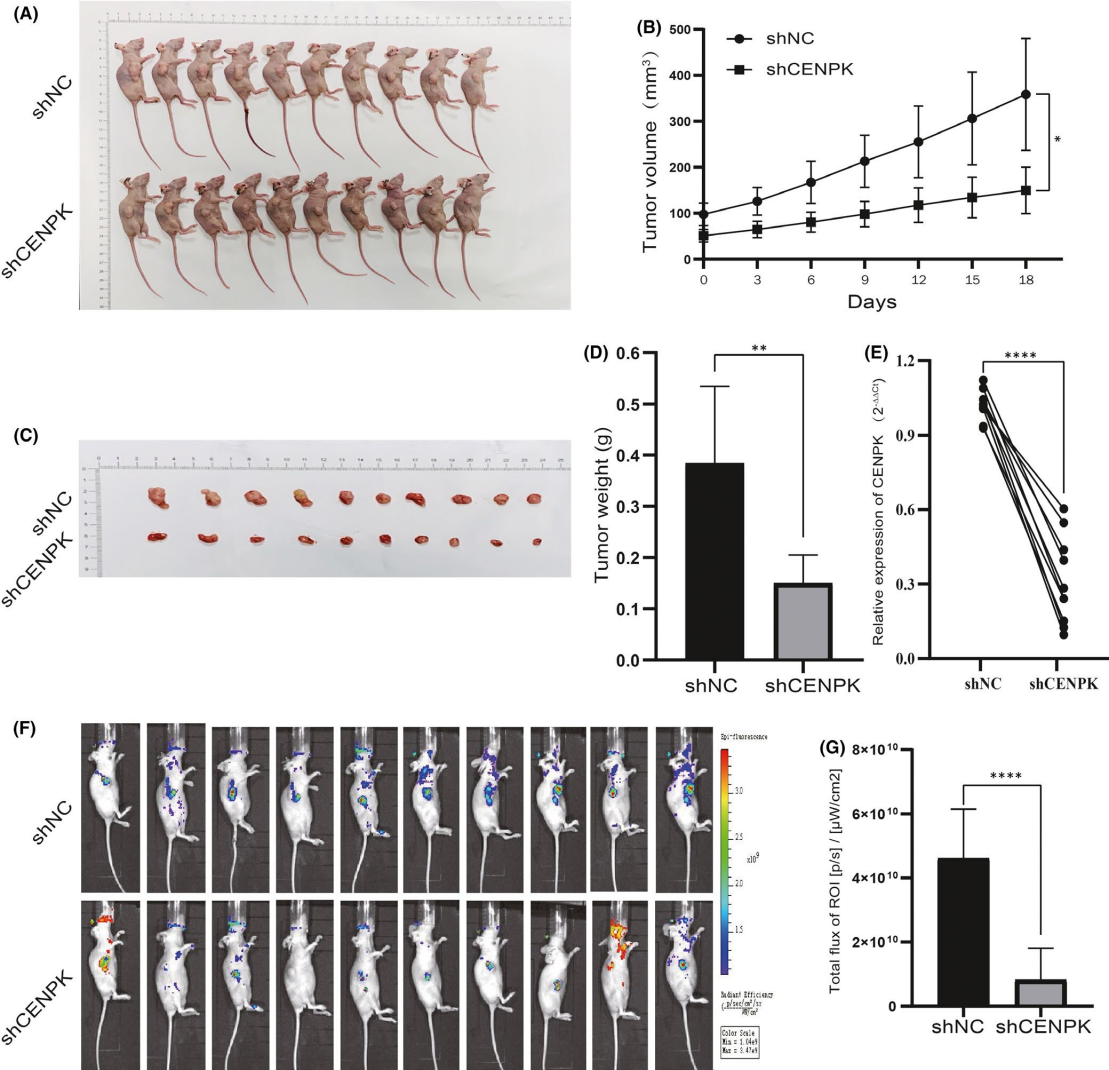
Knockdown of CENPK inhibited cell growth in vivo. A, AGS cells transfected with CENPK‐shRNA and control shRNA were injected into nude mice (*n* = 10), respectively. The photograph shows tumour formation in two groups. B, Tumour volumes and tumour formed (C) in shCENPK group were significantly smaller than that of shNC group. D, The shCENPK group had significantly lighter tumour weights at the end of the experiment than the shNC group. E, Analysis of the expression level of CENPK mRNA in xenograft tumours. F, Tumour formation was examined by bioluminescence assay in vivo. G, The total flux of the region of interest (ROI) in the shCENPK group was significantly lower than that of the shNC group. *p < 0.05, **p < 0.01, ****p < 0.0001

Next, we examined the effect of CENPK on GC growth in vivo. Further studies are needed to elucidate the potential effect of CENPK on GC formation in vivo. After construction of the subcutaneous GC xenograft model, the tumour sizes in the groups were recorded at set intervals during the growth phase. As shown in Figure 5A‐B, tumour volume in the CENPK‐silencing group was lower than that in the control group (*p* < 0.05), and the tumour weight in the shCENPK group was remarkably lower than that in the control group (Figure 5C‐D, *p* < 0.001). It was demonstrated that cell lines transduced with CENPK shRNA lentivirus in in vivo experiments can produce smaller tumours due to CENPK silencing. We then analysed the expression levels of CENPK mRNA in xenograft tumours and found that CENPK mRNA levels were significantly lower in the shCENPK group than in the control group (Figure 5E, *p* < 0.0001). In addition, in vivo bioluminescence was used to examine tumour formation. Notably, the fluorescence intensity of green fluorescent protein in the shCENPK group was weaker than that in the shNC group (Figure 5F). The ROI of the shNC group was significantly higher than that of the shCENPK group (Figure 5G, *p* < 0.0001). These results suggest that CENPK may influence gastric cancer growth both in vivo and in vitro.

### 3.8 GO and KEGG pathway enrichment analysis

**FIGURE 6:**
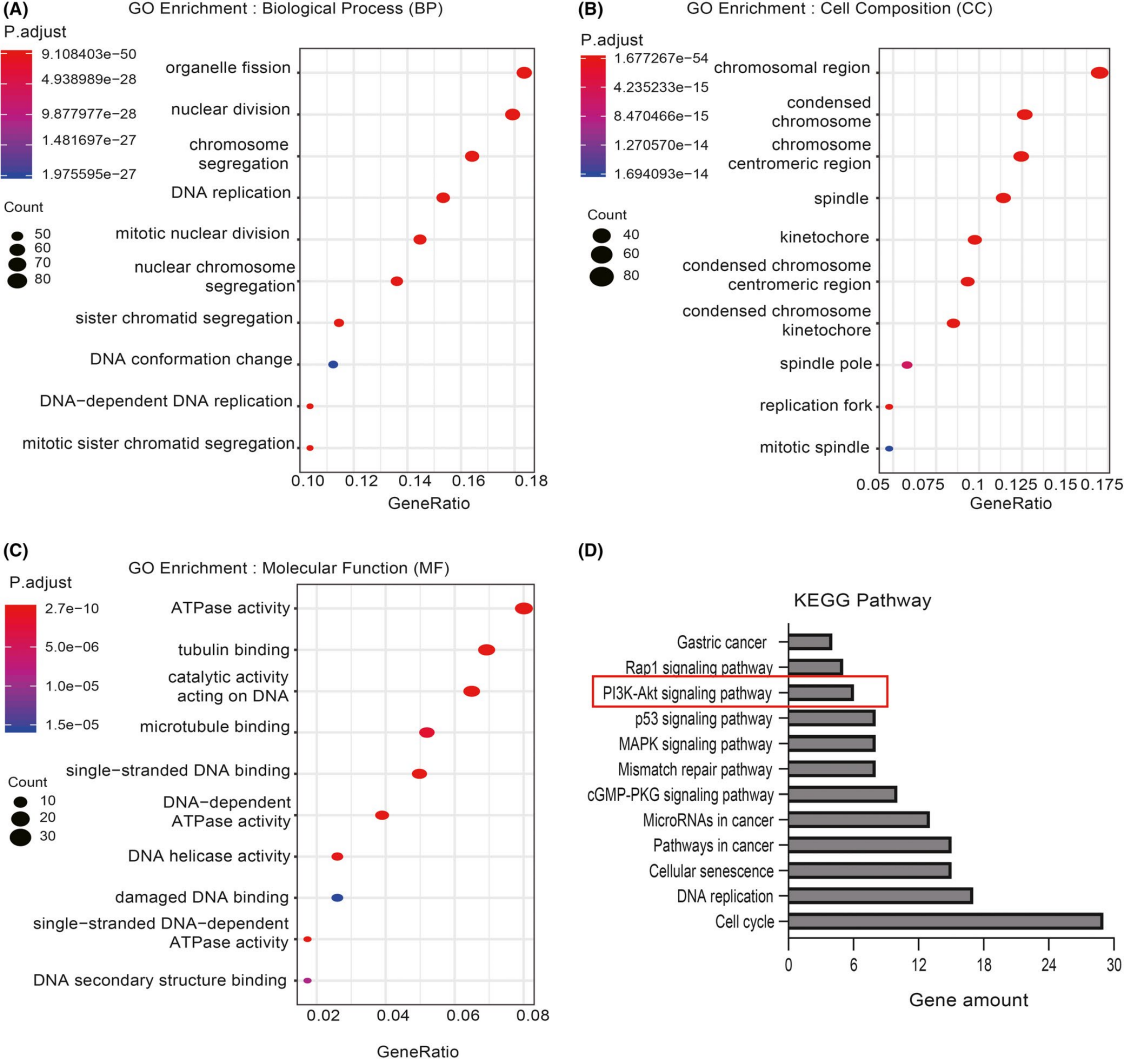
GO and KEGG pathway enrichment analysis. Top 10 significantly enriched GO annotations of biological process (A), cell composition (B) and molecular function (C). D, Top 12 significantly enriched KEGG pathways

To further explore the molecular mechanisms by which CENPK promotes GC, GC samples from TCGA were used for annotation. The top 10 results of GO enrichment analysis suggested that organelle fission, nuclear division and chromosome segregation were dramatically regulated by CENPK alteration in GC (Figure 6A). The chromosomal region, condensed chromosome, ATPase activity and tubulin binding were also significantly controlled by CENPK alterations (Figure 6B,C). Figure 6D shows the top 12 pathways in GC most relevant to CENPK as identified by KEGG enrichment analysis. For example, the PI3K‐AKT signalling pathway, DNA replication, cellular senescence, p53 signalling pathway, pathways in cancer and cell cycle were all enriched.

### 3.9 CENPK inhibits apoptosis and promotes cell growth involving the PTEN‐PI3K‐AKT signalling pathway

**FIGURE 7:**
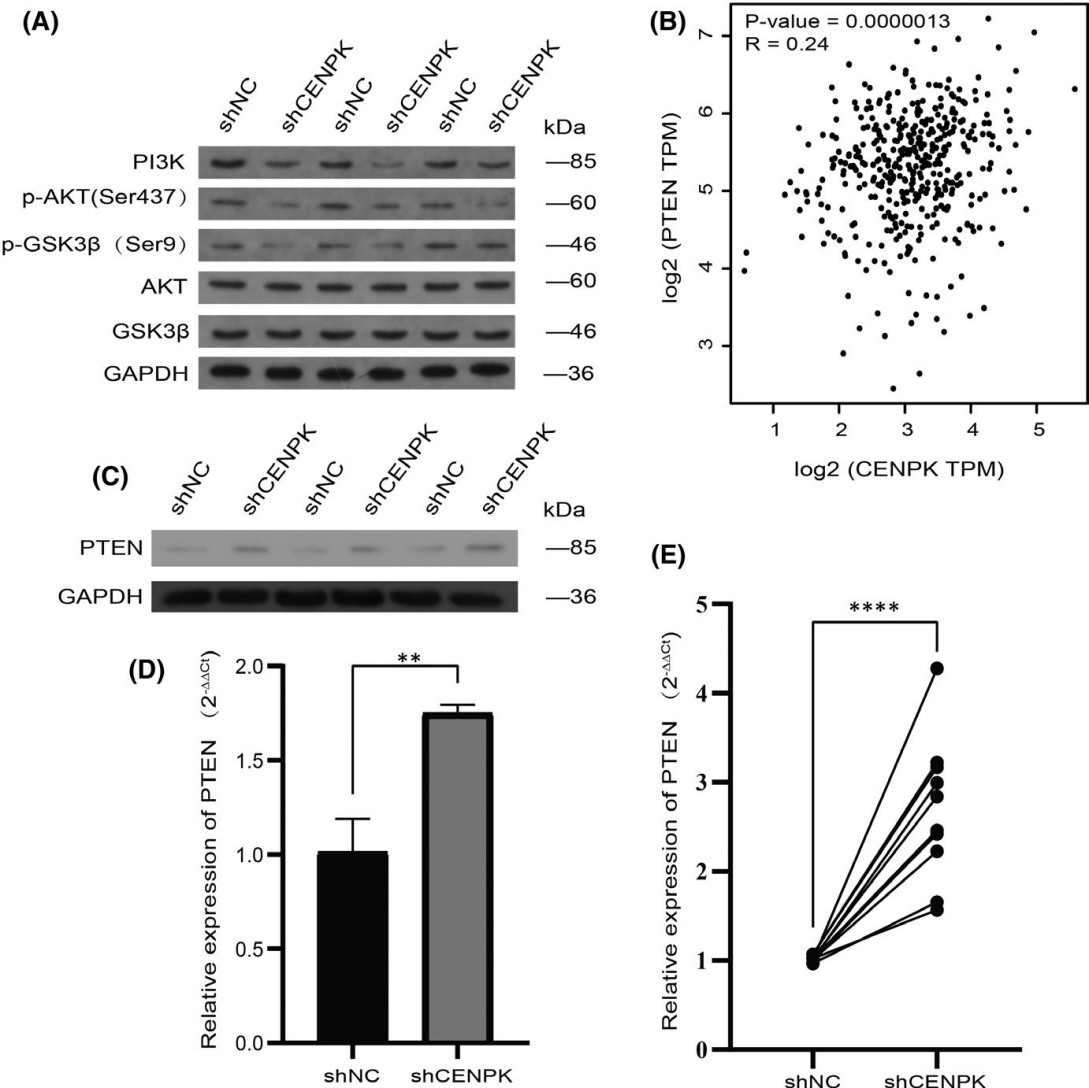
CENPK promoted cell proliferation and inhibited apoptosis involving PTEN‐PI3K‐AKT signalling pathway. A, The Western blotting assay indicated that the levels of proteins p‐Akt (Ser437), PI3K, and p‐GSK3β(Ser9) in the shCENPK group were significantly lower than that in the NC group. B, The correlation analysis was performed between CENPK and PTEN in TCGA gastric cancer samples by GEPIA online database. The levels of PTEN in AGS cells transfected with or without shCENPK were analysed by qRT‐PCR (C) and Western blotting (D). E, Analysis of the expression level of PTEN mRNA in xenograft tumours. **p < 0.01, ****p < 0.0001

Enrichment analysis results of GO and KEGG illustrated that CENPK is closely related to the cell cycle. Our data also indicated that CENPK silencing induced G1 phase arrest and promoted apoptosis in AGS cells. After shCENPK‐transfected AGS cells, the expression of p21 and p27 in the shCENPK group increased, while the expression of CDK4 and cyclin D1 decreased. KEGG enrichment analysis revealed that PI3K‐AKT was enriched, and the upstream signalling pathways of p21, p27, CDK and cyclin were PI3K‐AKT signalling pathways.^28^ Therefore, we speculated that CENPK might regulate the cell cycle via the PI3K‐AKT signalling pathway. As shown in Figure 7A, CENPK silencing reduced the expression of PI3K, p‐Akt (Ser437) and p‐GSK3β (Ser9), while the changes in AKT and GSK3β were not obvious. Hyperactivation of the PI3K‐AKT signalling pathway is often accompanied by a deficiency of PTEN.^29^, ^30^ Therefore, we used the Pearson correlation coefficient in GEPIA to detect the correlation between CENPK and PTEN in TCGA gastric cancer samples. As shown in Figure 7B, the correlation between CENPK and PTEN has a *p* value of 0.0000013 and an *R*‐value of 0.24. In the present study, it was also confirmed that after CENPK knockdown, PTEN mRNA and protein levels in shCENPK group were significantly higher than those in the control group (Figure 7C,D). Moreover, we examined the expression levels of PTEN mRNA in tumours of sacrificed animals. The shCENPK group had significantly higher expression levels of PTEN mRNA than the shNC group in xenograft tumours (Figure 7E, *p* < 0.0001). The results of the correlational analysis indicated that CENPK accelerated cell proliferation, interrupted cell cycle and influenced cell survival via the PTEN‐PI3K‐AKT signalling pathway in GC.

## 4 DISCUSSION

The occurrence of GC is a multi‐stage and multifactorial process, that severely threatens the health of Chinese people.^2^, ^31^ Therefore, it is urgent to explore new GC‐related molecules as potential therapeutic targets. Centromeres, large protein complexes that accumulate in the centromere region of chromosomes, promote chromosome separation during the cell cycle.^32^ Growing evidence indicates that dysregulation of centromere proteins is closely associated with tumorigenesis, and the family of centromere proteins has been reported to be overexpressed in various malignant tumours, including GC.^33^, ^34^ For example, a previous study showed that CENPO is upregulated in GC and is related to prognosis. Knockdown of CENPO inhibited cell growth and promoted apoptosis.^34^ However, the expression and function of CENPK in GC remain unknown. Collectively, our data from this study confirmed that CENPK is overexpressed in GC cell lines and tissues, and GC patients with high CENPK expression have a worse prognosis. Lentiviral‐mediated gene knockdown of CENPK suppressed GC growth in vitro and in vivo, induced G1 phase arrest and promoted apoptosis in vitro through the PTEN‐PI3K‐AKT signalling pathway.

Previously, CENPK was identified as an oncogene that was overexpressed in hepatocellular carcinoma, ovarian cancer and triple‐negative breast cancer.^15^, ^16^, ^17^ The OS of ovarian cancer patients with low CENPK expression was significantly longer than that of patients with high CENPK expression (*n* = 26).^15^ In the present study, we first identified that CENPK was markedly upregulated in GC using bioinformatics databases, further validated in GC cell lines and tissues. Moreover, the present study indicated that the OS of GC patients with high CENPK expression was considerably worse than that of patients with low expression and that CENPK expression was statistically correlated with microsatellite status and pathological stage T according to the TCGA database. Multivariate analysis showed that CENPK expression levels and clinicopathological characteristics, such as age, histologic grade, tumour stage and distant metastasis in GC patients were independent prognostic factors for OS. These results suggest that CENPK may be an oncogene in GC, which is consistent with the results of previous studies. Therefore, we speculate that CENPK plays a vital role in the occurrence of GC, which may be closely related to GC.

We confirmed that CENPK is overexpressed in GC cell lines and tissues. We also investigated the influence of CENPK silencing on GC cell lines. Consistently, AGS cell lines forced to knockdown CENPK exhibited limited proliferative capacity and were more prone to apoptosis. Tumour xenograft studies further demonstrated the function of CENPK in accelerating GC growth in nude mice. These studies demonstrated that CENPK had a stimulatory effect on GC. Our findings elucidate for the first time that CENPK is upregulated in GC and suggest that CENPK is an oncogene that promotes GC progression, which is consistent with the role of CENPK in other tumours, such as ovarian cancer,^15^ triple‐negative breast cancer^16^ and hepatocellular carcinoma.^17^ The present study also revealed that CENPK silencing blocked the cell cycle in the G1 phase. According to the cell cycle assay, we found that attenuated CENPK in AGS cells delayed the cell cycle process and arrested at the G1 phase. It is suggested that the abnormal expression of CENPK severely interrupts the cell cycle process.

CENPK was reported to have a decisive influence on the correct kinetochore function and, mitotic progression,^35^ and is associated with cell cycle and mitotic spindle assembly in bladder cancer.^36^ To further explore the molecular mechanism of CENPK in promoting tumorigenesis in GC, enrichment analysis of GO and KEGG based on the TCGA gastric cancer database was performed. The results revealed that molecular processes related to the cell cycle, such as tubulin binding, chromosomal region, chromosome segregation, nuclear division, condensed chromosome and organelle fission were significantly enriched. KEGG enrichment analysis revealed that the most relevant pathway to CENPK was the cell cycle in GC. Our results are consistent with the findings of Liu et al. in bladder cancer.^36^ The results of KEGG enrichment analysis also indicated that the PI3K‐AKT pathway was significantly enriched. Accumulating evidence suggests that the PI3K‐AKT signalling pathway is involved in the pathogenesis of GC and regulates cellular processes, such as differentiation, proliferation and metastasis.^37^, ^38^, ^39^ Moreover, inhibition of the PI3K‐AKT signalling pathway can block the cell cycle in GC cell lines.^40^ Therefore, we focussed on the PI3K‐AKT pathway. Our results showed that CENPK silencing decreased the expression of PI3K, p‐AKT(Ser437) and p‐GSK3β(Ser9) in GC cells. CENPK knockdown may promote apoptosis and inhibit cell proliferation through the PI3K‐AKT signalling pathway in GC. Unfortunately, there were no rescue experiments to further confirm this finding. Previous evidence indicated that the activation of the PI3K‐AKT pathway in GC was accompanied by reduced expression of PTEN.^41^ Phosphorylation of PTEN can reduce the activation of AKT and hamper all downstream signalling events regulated by AKT.^42^ PTEN malfunction is closely associated with aberrant activation of the PI3K‐AKT pathway. AKT activation due to PTEN deficiency is strongly associated with the occurrence and development of GC.^43^ Bioinformatics analysis suggested that CENPK and PTEN were strongly correlated in GC. Our results revealed that CENPK silencing elevated PTEN expression in vitro and in vivo. Collectively, CENPK facilitated proliferation and inhibited apoptosis through the PTEN‐PI3K‐AKT signalling pathway.

## 5 CONCLUSION

In summary, the present study indicated that CENPK expression is upregulated in GC and is associated with the prognosis of GC. CENPK silencing inhibits GC cell proliferation in vivo and in vitro by promoting apoptosis and blocking the cell cycle at the G1 phase. Additionally, CENPK may regulate cellular behaviours through the PTEN‐PI3K‐AKT signalling pathway in GC. In conclusion, our results illustrate the potential role of CENPK in promoting GC and suggest that CENPK may be a potential target for intervention in GC.

## CONFLICT OF INTEREST

The authors confirm that there are no conflicts of interest.

## AUTHORS CONTRIBUTION

**Shusheng Wu:** Conceptualization (lead); Data curation (lead); Formal analysis (lead); Investigation (lead); Methodology (lead); Software (lead); Visualization (lead); Writing‐original draft (lead); Writing‐review & editing (lead). **Lulu Cao:** Investigation (lead); Project administration (lead). **Lihong Ke:** Methodology (equal); Validation (equal). **Ying Yan:** Investigation (equal); Software (equal). **Huiqin Luo:** Methodology (supporting); Visualization (equal). **Xiaoxiu Hu:** Data curation (equal); Methodology (supporting). **Jiayu Niu:** Methodology (supporting). **Huimin Li:** Methodology (supporting); Supervision (equal). **Huijun Xu:** Conceptualization (supporting); Methodology (supporting). **Wenju Chen:** Conceptualization (supporting); Methodology (supporting). **Yueyin Pan:** Funding acquisition (equal); Supervision (equal). **Yifu He:** Conceptualization (equal); Funding acquisition (equal); Project administration (equal); Supervision (equal); Writing‐review & editing (equal).

## Supporting information

Table S1

Table S2

## Data Availability

The data that support the findings of this study are available from the corresponding author upon reasonable request.
